# A Multimodal Analytics Platform for Journalists Analyzing Large-Scale, Heterogeneous Multilingual, and Multimedia Content

**DOI:** 10.3389/frobt.2018.00123

**Published:** 2018-10-29

**Authors:** Stefanos Vrochidis, Anastasia Moumtzidou, Ilias Gialampoukidis, Dimitris Liparas, Gerard Casamayor, Leo Wanner, Nicolaus Heise, Tilman Wagner, Andriy Bilous, Emmanuel Jamin, Boyan Simeonov, Vladimir Alexiev, Reinhard Busch, Ioannis Arapakis, Ioannis Kompatsiaris

**Affiliations:** ^1^Information Technologies Institute, Centre for Research and Technology Hellas, Thessaloniki, Greece; ^2^High Performance Computing Centre, University of Stuttgart, Stuttgart, Germany; ^3^Department of Information and Communication Technologies, Pompeu Fabra University, Barcelona, Spain; ^4^Catalan Institute for Research and Advanced Studies, Barcelona, Spain; ^5^Deutsche Welle, Bonn, Germany; ^6^Everis, Madrid, Spain; ^7^Ontotext Corp, Sofia, Bulgaria; ^8^Linguatec, Munich, Germany; ^9^Telefonica, Madrid, Spain

**Keywords:** multimodal analytics platform, journalism, multilingual content analysis, big data, knowledge extraction, semantic analysis

## Abstract

Analysts and journalists face the problem of having to deal with very large, heterogeneous, and multilingual data volumes that need to be analyzed, understood, and aggregated. Automated and simplified editorial and authoring process could significantly reduce time, labor, and costs. Therefore, there is a need for unified access to multilingual and multicultural news story material, beyond the level of a nation, ensuring context-aware, spatiotemporal, and semantic interpretation, correlating also and summarizing the interpreted material into a coherent gist. In this paper, we present a platform integrating multimodal analytics techniques, which are able to support journalists in handling large streams of real-time and diverse information. Specifically, the platform automatically crawls and indexes multilingual and multimedia information from heterogeneous resources. Textual information is automatically summarized and can be translated (on demand) into the language of the journalist. High-level information is extracted from both textual and multimedia content for fast inspection using concept clouds. The textual and multimedia content is semantically integrated and indexed using a common representation, to be accessible through a web-based search engine. The evaluation of the proposed platform was performed by several groups of journalists revealing satisfaction from the user side.

## Introduction

The rapid expansion of information technologies and the low cost of recording media have made available large amounts of multilingual and multimedia content. TV, radio, newspapers, blogs, and social media are the main means of dissemination of this Big Data content worldwide. Within this context, all media companies have made significant efforts to embrace the new sources that have risen in the last decade and have thus tried to integrate social media into their workflows and output. However, the plethora of sources, combined with the language barriers restrict journalists to limited media resources, leaving the population in each of any encapsulated area in its own “filtered bubble”—without the realistic opportunity to understand the perspective developed in another area or country.

To break this isolation, the development of new technologies is required with innovative and effective functionalities to provide integrated access to multilingual and multicultural news articles across nations, to ensure its context-aware, spatiotemporal, semantic interpretation, and to correlate and summarize the collected content into a coherent whole. In particular, these technologies should capture, interpret and relate various subjective views of news content as generated by TV, radio, newspapers, blogs, and social media.

As far as journalists are concerned, they are mainly interested in spotting stories or angles that may have escaped the attention of their competitors. Moreover, journalists aim also to find and understand the level of audience interest in a particular story, gather and communicate the news in text, video, or audio. Internal findings from interviews and questionnaires of expert users have shown that journalists require support in the user requirements analysis, see for example^1^:

- Summarizing the content of heterogeneous, complex, and lengthy text documents.
- Distilling the most significant information of the original source document.
- Language-agnostic interpretation and understanding of the specific meaning of a textual document.
- Linking and retrieving content which is relevant to their current interests or needs
- Combining textual data with other related multimedia items into unified multimodal objects.
- Extract knowledge about how a story, such as a news event or a promotional campaign, varies over time.
- Filtering open online discussions, which are relevant to the journalist's interest and goals.

Although there are several research works focusing on these disciplines independently, there is a gap in accessing these large and heterogeneous online resources in a holistic manner. In this paper, we propose a unique platform for journalists integrating multimodal analytics techniques, crawling and indexing of multilingual and multimedia information from heterogeneous resources, text summarization, and translation into the language of the journalist, named entity, and concept extraction from textual and multimedia content for fast inspection using word clouds, and semantic analysis of the textual content. The proposed platform has been positively evaluated by several groups of journalists, as presented in this work.

Section Related Work discusses relevant platforms, systems, tools, and frameworks which are relevant and useful for a journalistic use case scenario. Section The Proposed Framework presents our framework, where we list some functionalities and requirements than need to be met. Sections Harvesting Layer, Distillation Layer, and Delivery Layer describe the three layers of the proposed platform, namely the harvesting, the distillation and the delivery layers, respectively. These layers present the data collection process, high-level feature extraction, as well as details about the functionalities of the system that are involved in the content extraction and analysis. In section Infrastructure we present system-centered and user-centered evaluation of the platform.

## Related work

Platforms that offer analytics to journalists have been presented in the literature. Carvalho et al. (2017) present the so called “MISNIS,” which is a platform for international broadcasters. The platform collects Twitter data in Portuguese language and visualizes geolocated tweets, estimates user influence in the graph of mentions using the PageRank centrality measure, performs sentiment analysis and topic detection. Contrary to this approach, we present a language-agnostic approach that is also multimodal and is able to analyze multimedia content, summarize its text and its extracted concepts from multiple online sources.

A hashtag recommendation system that is able to capture the evolution of news items and associated hashtags is presented in Shi et al. (2018), aiming to track an event for a journalistic scenario. NavigTweet (Francalanci and Hussain, 2017) is a platform that is able to explore and visualize the influence-based Twitter network. The platform delivers analytics which are based on the metadata of the tweets and shows them in a User Interface. User-based evaluation rates NavigTweet based on qualitative criteria. In Diakopoulos et al. (2010), Twitter messages corresponding to events were analyzed in event reasoning, visualization, and analytics. Their visual analytics tool, namely “Vox Civitas,” is designed to support journalists and media professionals in extracting news from large scale aggregations of social media content. The involved technologies for content analysis are sentiment, relevance, uniqueness, and keyword extraction. However, our platform includes additional functionalities, modalities, and sources of information, and is not restricted to the analysis of the textual modality. Surveys in event analysis and visual analytics frameworks from social multimedia data can be found in Liu et al. (2016) and Wu et al. (2016), respectively, with a particular focus on the methods for the development of each functionality.

The in-depth market analysis done on media monitoring tools revealed that there were no other existing tools with all the envisioned features, which led us to separate the research into different sections in order to keep the analysis comparable. The categories cover among others Social News Aggregators, such as Virato^2^, Social Network Search and Analysis, such as Topsy, different online media monitoring services, such as Newsexplorer^3^, as well as tools for text analysis, extraction and comparison, such as Semantic Wire. In the area of Social News Aggregators, tools like Virato or Rivva^4^ help people by showing them the most discussed topic currently on the web. Virato is a paid-for service; it focuses on the analysis of the most widely shared articles in social networks. Rivva, on the other hand, is a freely available tool, open to use for everyone, listing the most popular articles from German media and highlighting the number of their shares in social networks. Topsy is a social media search tool with focus on Twitter that allows users to search for keywords and lists findings from Twitter in multiple languages, including graphical analysis over time. The aforementioned categories are examples that were considered in the design of the proposed platform.

We present tools that target to classic media monitoring for the online world, due to the fact that our focus is not only on social networks, but also on the internet in general. This leads us to another category, the Online Media Monitoring Services, where there are systems like mention^5^ (now called alert.io) with similar functionalities to Google alert. A keyword-based search on the web is used to notify the user about every hit they find, including social media. Similar tools are Brandwatch^6^ which is used to monitor the competition in any business sector. Both options are relevant to our work, due to their crawling procedures, the visualization of their results, and their in-depth analysis.

Regarding tools and APIs whose goal is deeper text understanding, some examples are *churnalism*, opencalais^7^, and AlchemyAPI^8^, having as main requirement the Natural Language Processing (NLP) paradigm. Opencalais has been developed further, now offering a better tagging system to extract entities, topic codes, events, relations, and social tags. AlchemyAPI has extended its features, covering now also face detection, celebrity name-, age range-, and gender recognition, as well as face position. However, the proposed platform goes beyond state-of-the-art tools as it integrates multimodal analytics, indexing of multilingual and multimedia information, text summarization and translation, named entity and concept extraction, and semantic analysis into one unique platform. A general comparison overview is shown in Table 1.

**Table 1:** Functionalities of tools currently available.

	**Automatic Language Detection And Translation**	**Multiple Source Integration**	**Analysis**			**Entity Detection**	**Categorization**	**Enrichment**	**Automatic Summarization**
			**Semantic**	**Text Structure**	**Network**				
Social News Aggregator	NO	Twitter, Facebook, G+, Blogs	Partly	Partly	YES	NO	YES	NO	NO
Network and Search analysis	NO	Twitter only	NO	NO	Partly	NO	Partly	NO	NO
Online MM services	YES	Social media and the web (to different extents)	NO	NO	Partly	Partly	Partly	Mainly not	NO
Text analytics, extraction and web news filter	Partly	Mainly manual input necessary	Mainly not	Partly	Mainly not	YES	YES	NO	NO
Social Networks	YES	NO	Partly	Partly	Partly	Partly	Partly	Partly	NO

A major change in this game is the social network analysis. Both Facebook and Twitter offer built-in machine translation in their User Interfaces. Specifically, networks, including some of their competitors/subnetworks (e.g., Instagram), also serve trending topics and in-depth search features to help users grasp the large amounts of data coming through these channels. Some of these efforts are fairly new, others have been around for a while, but are constantly improving user service, e.g., through artificial intelligence (AI) and machine learning (ML). There is no generic solution available, even though companies like Hootsuite are trying to become the one-for-all solution, building on top of the networks' APIs.

Contrary to the aforementioned approaches, our unified platform integrates for the first time all functionalities described in detail in sections Harvesting Layer, Distillation Layer, and Delivery Layer. The novel platform is able to mine and distill content of unstructured heterogeneous and distributed multimedia data in multiple languages, including semantic analysis and reasoning. User- and context-based analysis of multilingual content is also provided by analyzing content from the user perspective to extract sentiment and context. In addition we analyze computer-mediated interaction in the web and specifically in social media. In the following section, we present our framework on which we base the design of our platform.

## The proposed framework

The proposed platform's capabilities can be summarized as follows:

- **Use of different media sources:** collecting news and multimedia content from multiple news sources including news sites, social media, and online media which involve aggregating the content from the diverse sources and storing in into a central repository for analysis.
- **User-oriented queries and user-defined search:** requesting for (predefined) online sources according to users' interests using keywords, ability to filter sources, media types, according to language, location, date and time.
- **Automatic language detection and translation:** detecting and translating textual content and speech (audio transcripts) from an unknown language to the language of the user.
- **Automatic summarization:** creation of a summary with the most important clues of a given text document, social media threads, or audio-visual content.
- **Named Entities Extraction:** extracting named entities (i.e., author name, names, brands, geo-location) from text and audio for further analysis.
- **Enrichment**: linking textual data with related multimodal data to identify common characteristics between different news items (e.g., topics, events), adding related social media content, clustering similar items, and realizing speech-to-text analysis of audio files.
- **System that is easily configurable by the user and that is easy to use via a self-explainable GUI:** developing a platform that can be easily configured by the user, that allows filtering, saving of results, combining results and that provides several types of visualization of the information in multiple languages.

While functional requirements capture the system's behavior, we consider valuable feedback and constraints from stakeholders need to be respected. The platform has to be service-oriented, standardized, interoperable, scalable, reusable, sustainable, and simple. Firstly, it should be created as an orchestration of discrete functionalities, available as services to the system. Secondly, it has to be serialized and exchangeable using open, and well-established formats and standards. Thirdly, each functionality should be exposed a set of services, while the underlying technology of each service can change without affecting the rest of the system. Fourthly, the platform has to be developed in a way that can deal with large amounts of data and users. Moreover, discrete functionalities have to also be available as standalone services and to be reusable. Furthermore, the services of the platform should be executed on a geographically distributed infrastructure, allowing also an iterative or evolutionary development toward a sustainable architecture. Finally, above all, the platform architecture has to be user-friendly and easily understood, in terms of processes and topology.

Therefore, we propose a dynamic system, designed to generate new intelligence from large streams of online multimodal content, monitoring media sources, and social networks. The platform hosts two interconnected subsystems: the offline and the online one.

The offline subsystem is asynchronous and is responsible for the collection and the analysis of all available content being stored in the Central News Repository (CNR). The Content Extraction Pipeline (CEP) can process multilingual textual and multimedia data from CNR. The services included in the CEP are the language detection, Named Entity recognition, and concept extraction services, the extractive summarization service, the classification service, the context extraction service, the audio extraction, and automatic speech recognition (ASR) service, as well as the concept and event detection service. These services will be described in separate sections below.

The online subsystem is the connection between the user interfaces and the applications that sit on top of the backend and it includes the following services: the topic-event detection service, the similarity search, and the machine translation.

**Figure 1:**
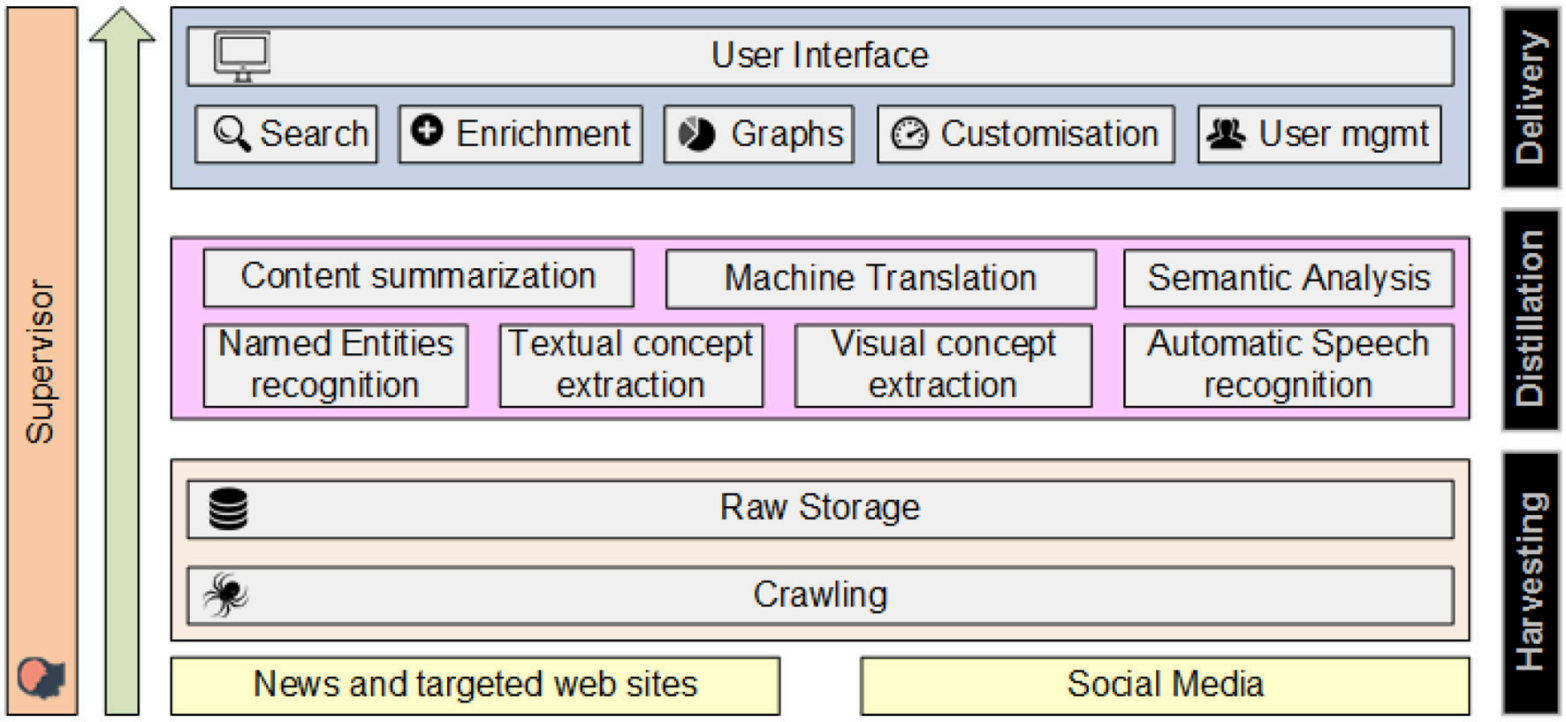
The architecture of our framework.

The proposed architecture of the platform (see Figure 1) aims at satisfying the user requirements detailed above and is constituted of three major subsystems that correspond to three steps in the value generation process: the Harvesting layer, the Distillation layer, and the Delivery layer. In addition, the Supervisor component integrates the layers and orchestrates the data flows and execution of all services. Specifically, the Harvesting layer captures and aggregates cross-language and multimedia content from a variety of sources into the proposed platform, for further analysis. The Distillation layer constitutes of a set of processes, which analyze the data collected and indexed from the harvesting layer, so as to generate high-level knowledge from it. Analytical processes include linguistic analysis, inference, multi-dimensional clustering, and summarization. The Delivery layer is a set of user interfaces, offering interaction with the platform and understanding of the content. In the following three sections we present each layer and module/service in detail.

## Harvesting layer

The purpose of the developed crawling infrastructure is to facilitate content extraction from heterogeneous resources (e.g., news articles, data from financial portals) and the retrieval of web data (e.g., blogs, social media). Data are retrieved from two sources, namely Twitter, and web pages.

### News articles crawler

The news crawler discovers and downloads recent news articles in a predefined set of sources (websites) by applying a modular architecture, integrating big data processing technologies, such as Hadoop, HBase, Nutch, and Solr. The crawling component, Apache Nutch^9^, forms the core of the news crawler's architecture and, initially, reads URLs from a priority queue and downloads the corresponding identified web pages, then parses the content of the collected pages to discover new URLs, and finally store the web pages in a data store. Crawling is available through MapReduce^10^ jobs executed on the Apache Hadoop^11^. Regarding the collected webpages, we employ the Apache HBase^12^ data store.

### Social media crawler

As Twitter collector, the Stream Manager from the SocialSensor^13^ EU Project is used. It includes a variety of APIs that collect content relevant to a keyword, a user or a location from a set of social streams including Twitter, Instagram, and Facebook. The Twitter collector gathers Twitter posts for a set of high-activity hashtags that are pre-specified. These collected posts, and the information regarding the author (social media user) such as the associations (e.g., mentions, follows) found inside the posts are then stored in a MongoDB database. The associations are not only user-to-user relations, but also involve user-to-webpage or post-to-webpage associations that reveal information for the journalistic usage and news article authoring scenarios.

## Distillation layer

The presentation of modules is given into two dimensions that are related to the type of input they accept. Thus, there is the textual dimension, which includes all modules that process textual data and the multimedia dimension, which includes modules handling multimedia data.

### Textual dimension

#### Content summarization

The basic types of multilingual (English, French, German, and Spanish) document summaries provided by our platform include: an extractive summary, which consists of the most relevant statements (usually sentences) extracted from the original document, and c) an abstractive summary, which is a text gist of the content extracted from a document or a document collection, produced using Natural Language Generation (NLG) techniques.

The extractive summarisation module uses the summarization toolkit SUMMA^14^, which is embedded into the GATE framework^15^ for Natural Language Processing. SUMMA provides resources and tools for building single and multiple document and multilingual extractive text summarization applications that use statistical measures to determine the relevance of a sentence in a document to the summary (including the relative position of the sentence in the text, the part it appears in (title, introduction, main body, conclusions), tf-idf values of its tokens, etc.

The abstractive summarization module contains three main submodules: language analysis, text planning, and linguistic generation. The **language analysis** module performed by a text analysis pipeline that requires as input the textual part of a news article or any collected text, in a given language. This content is first analyzed and represented as a forest of Deep-Syntactic Structures (DSyntSs) (Ballesteros et al., 2016). If the given language differs from English, we map every lexeme in DSyntSs into an English lexeme using bilingual dictionaries. Thus, we arrive at an “interlingua” structure with neutral language representations. These “interlingua” structures are then mapped onto ontologies, modeled as RDF triples, enriched with Frames from the FrameNet lexicon (Fillmore et al., 2002), and stored in a semantic repository. **Text planning** assesses the relevance of each semantic structure with respect to the topic of interest of the user (based on semantic entity co-occurrence) and orders the relevant structures in order to ensure coherence between them. Our text planning approach involves the exploration of the available contents and their evaluation based on some empirical relevance metric. More specifically, a graph view of the contents in the semantic repository is adopted and new contents corresponding to user-specified entities, for which a summary is to be generated, are incrementally searched and assessed according to a semantic similarity metric based on distributional sense vectors. **Linguistic generation** initially transfers the lexemes from the semantic repository to the user-defined target language, exploiting existing multilingual lexical resources. We then determine the structure of each sentence and all words are inserted and linked with pre-defined relations (Mille et al., 2016).

#### Machine translation

Automatic machine translation (MT) is incorporated into our platform to translate the summarized output as well as to be involved in the multilingual content analysis pipeline. Given that the covered languages are English, German, Spanish, Bulgarian, and French, and thus the language pairs created were Spanish, French, German, and Bulgarian into English and vice versa. Today, the most common approach for the development of machine translation (probabilistic-based) systems is the use of statistical techniques. This implies the availability of parallel corpora with texts in two or more languages that are perfect (human) translations of each other. Corpora collection targets both bilingual and monolingual corpora. Bilingual corpora are used for tuning and testing purposes, while monolingual corpora are used for language modeling, normalization, and pre-processing. The MT system was trained and tuned (Bertoldi and Mederico, 2009) on both freely available open source general domain corpora, such as DGT's translation memories, European Parliament speeches, and the JRC-ACQUIS corpus (Tyers and Alperen, 2010).

As far as pre-processing is concerned, a bilingual crawler was adapted that uses seed URLs (Kaumanns, 2011). The crawler looks for pages with identifiable language flags, and for each found webpage, it looks for a corresponding page in another language. Then, it stores the retrieved HTML files and processes them offline using a special HTML-Parser. The effective validation of word sequences and assignment of higher probabilities to most plausible ones in the process of language modeling, the language model (LM) construction module builds probabilistic N-gram language models, by using the SRILM tool (Stolcke, 2002).

#### Named entities recognition

Named Entities (NE) are words such as “London,” “Siemens” which are uniquely identified objects, or ambiguous names that accommodate multiple meanings, like “Barcelona,” pointing either to a city or to a region. The Named Entity (NE) recognition module identifies persons, locations, organizations, amounts and dates, as possible types of entity. The NE recognition module has two parts: first, a lookup for entities in a lexicon and second, additional identification for entities which do not belong to the lexicon.

The NE recognition procedure requires two resources: an NE lexicon and a NE recognition grammar. NE lexicons are organized along language and task, namely different lexicons handle different languages and different **lexicons** cover different tasks. Furthermore, for domain adaptation and accuracy improvement, text analytics are used, and the domain-specific names were added to the lexicon. Regarding task-related lexicon, there are the name lexicons that contain lexicalized named entities selected based on their appearance frequency in order to limit their size, indicator lexicons for storing the indicators of entity types and domain lexicons which are tuned for a particular domain and which are developed from domain-specific resources. NE **grammars**, being phrase-structure rules, augmented by scoring information, are used for detecting Named Entities and their assignment to their corresponding type. They consist of an identifier, a phrase structure part, a probability score, a feature handling section, and a comment field. The grammar distinguishes between NEs and non-NEs, provides a special rule set for lexicalized NEs (and their multiword parts), and identify possible NEs from patterns. Grammar also aids in the identification of unknown entities which are found in the text and their type assignment. The languages covered in the current version are English, German, Spanish, Bulgarian, and French.

#### Textual concept extraction

Concept extraction aims at identifying into textual documents all concepts that are of interest for the journalists and are modeled in the ontologies and datasets. The mentions to entities detected by the NE recognition module include mentions to specific individuals, organizations, places/locations, and time periods. The concept extraction module aims at extending the recognition to include references to domain-specific ones and thus vocabularies, ontologies, or datasets specific to the scenario of interest are required. The core of the proposed approach is to effectively identify mentions to entities in the domain-specific datasets, as specified by a journalist. The process of concept extraction follows the following steps: (1) Term-candidate detection, (2) statistical feature determination, (3) concept identification, and (4) combining different sources.

The module takes as input tokenized sentences of a document. Tokens are then lemmatized and annotated with Part of Speech and syntactic dependencies. The next step is the statistical feature determination where each term candidate is scored in order to indicate its termhood (that is, how likely it is that the term is a concept) and domain pertinence (to measure if a term is a general domain concept or specific to the domain under study). The statistical features implemented were the C-Value measure (Frantzi et al., 1998) and the Weirdness metric (Ahmad et al., 1999). The BabelFy concept identification step follows, that links and disambiguates among entities. The final step involves the combination of different sources given that at this point of the process, we have two lists of concepts: the list obtained by the statistical pipeline and the list generated by BabelFy. The terms generated by the statistical pipeline with a DomWeight below 0.8 or nested terms with a lower C-Value than the one of the term they belong to which are not found as not nested are filtered out. The remaining terms are sorted by decreasing C-Value and, when there is a tie, by DomWeight. The combined list is produced by intersecting the two individual lists. The code for the concept extraction module is provided at https://github.com/talnsoftware/concept_extraction.

#### Topic and event detection

Two levels of separation are provided for the document collection. Firstly, the module categorizes a news item into one of the six categories recognized by the IPTC news codes^16^, with the guidance of the journalists: “Health,” “Nature & environment,” “Science & technology,” “Economy & finance & business,” “Lifestyle & leisure,” and “Politics.” Secondly, the module clusters the given streams of news articles into more specific topics.

##### Category-based classification

The topic-classification module classifies news articles into predefined categories by considering solely textual information, given that the experiments showed that it is more reliable than visual information and that the existence of images inside news articles is not guaranteed. Specifically, the framework proposed fuses multiple textual features and applies Random Forests (RF) machine learning method (Breiman, 2001) to effectively classify news articles.

The two types of textual features used for representing suitably each news article are: (a) Word n-gram textual features, and (b) word embeddings. As far as n-grams are concerned, they are continuous sequence of *n* words taken from a body of a text document. The number of most frequent unigrams, bigrams, trigrams, and four-grams are 100, 50, 30, and 15, respectively. As far as word embeddings are concerned, Mikolov et al. (2013) proposed novel architectures and models for producing them. In particular, the Continuous Bag-of-Words (CBOW) and the Skip-gram models were introduced, known as word2vec, using the context of the words, i.e., the neighboring words in a sentence (within a large corpus). In our framework, we opted for the Skip-gram models, which given a word the model tries to predict the context of a word. The superiority of word2vec representation over simpler methods has been examined and shown in similar problems (Ju et al., 2015; Lilleberg et al., 2015).

The textual representation model (word2vec, n-grams) is the input of the classification model. The basic notion of the methodology is the fusion of all representations for the construction of a multitude of decision trees. We aggregate the outputs of the trees as constructed by the RF for the prediction of an unknown instance, i.e., a newly generated text document. We first train a separate Random Forest for each representation, and we then apply a late fusion strategy (Liparas et al., 2014) by computing weights for each representation's RF outputs. We use the Out-Of-Bag (OOB) error estimates for the computation of the modality weights. For each class and for each feature RF model, the corresponding OOB accuracy values are computed. These values are normalized and serve as weights for the RF models'. The code is available online here: https://github.com/MKLab-ITI/category-based-classification

##### Topic-event detection

Topic-event detection in news articles addresses the grouping of text documents that discuss about the same topic or event. Within our proposed platform, topic-event detection is a very important problem for journalists due to their need to efficiently detect articles, relevant to their needs, within a pool of news items produced regularly, given the unknown topics' number or labels. We employ a hybrid clustering methodology for topic detection that combines the popular Latent Dirichlet Allocation (LDA) with the DBSCAN-Martingale (Gialampoukidis et al., 2016), which is a density-based clustering method. It first estimates the number of topics (clusters) in a given collection of textual documents and LDA assigns news items into topics. The motivation for this combination is that LDA performs well on text clustering but requires as input the number of topics, while density-based clustering algorithms do not require the number of clusters, but their performance in text clustering is limited, especially when compared to LDA. The code is available online here: https://github.com/MKLab-ITI/topic-detection.

### Multimedia dimension

#### Concept extraction

In section Textual Concept Extraction we described the extraction of concepts as they are identified in textual content. In this section we describe the detection of visual concepts in multimedia files, such as videos and images from webpages and social media posts. The concepts targeted were proposed by journalists and include logos, concepts and objects. Specifically, the following concepts are handled: EnBW logo, E-On logo, Nuclear energy logo, RWE logo, Vattenfall logo, Outdoor factory smoke, Wind turbine, Solar panel, Lattice tower, Construction workers, People protesting, Speaking to camera, Fire and, Airplane flying. The representation of the media item is the first stage of the analysis and the classification stage follows.

##### Representation

The multimedia concept extraction module is applied onto the collected videos and images. **In case of a video**, we first extract the key frames out of the video for its representation, where we follow a temporal segmentation approach, shot segmentation that involves video partitioning into consecutive frames called shots and extracting keyframes from each shot. The procedure involves the use of the ffmpeg library^17^ for extracting the video's frames, the clustering of the extracted frames into shots following proposed by Tsamoura et al. (2008). In the shot segmentation procedure, we use Support Vector Machines (SVM) classifiers, and we finally select the middle frame as keyframe for representing the specific shot. **In case of an image** or a video keyframe, feature extraction refers to the extraction of visual features that represent each image or keyframe as a vector. Our concept extraction module is based on a Deep Convolutional Neural Network (DCNN) which is pre-trained using the Caffe^18^ tool in the work of Markatopoulou et al. (2016). The network was trained according to the 22-layer GoogLeNet^19^ architecture on the ImageNet “fall” 2011 dataset for 5055 categories (Russakovsky et al., 2015). The second last fully connected neural network layer of the second auxiliary classifier was used as a global image representation with a dimensionality of 1024.

The **classification** stage involves the assignment of concepts to the extracted images and video keyframes, using the extracted visual features. One SVM classifier is trained per each one of the desired classes (concepts), so as to assign a score that indicates the belief of each model that the corresponding concept appears in the image or video shot. The library used for implementing the classification models is LIBSVM^20^. With respect to the kernel type, the linear SVM version was chosen and regarding the gamma and C parameters, their default values (as provided in LIBSVM) were applied. Moreover, class weights were adjusted to be inversely proportional to their frequencies, because it is important to train classifiers capable of classifying even the least frequent classes. In the case of videos, and given that the training is made on keyframe level, during the test phase, in order to get the detected concepts/events from an unknown video, we get the prediction scores on keyframe level and we consider that if the concept appears in at least one keyframe, then the video contains this concept as well.

#### Automatic speech recognition

In this section we describe the automatic speech recognition (ASR) module, which transforms speech signal to text. The majority of ASR tools apply statistical approaches are based on Hidden Markov Models (HMMs), but differ in feature extraction, decoding, and acoustic/language modeling. Regarding the vocabularies used, the system adopts an open-vocabulary framework (Hahn and Rybach, 2011). This approach allows for recognizing unknown words based on sub-word units, and is far more flexible compared to the closed vocabulary approach, in which recognition is static and limited only to the words defined in the vocabulary. The procedure for the creation of recognition lexicons and language models, which are hybrids between whole words and sub-words, such as compound parts, morphemes, prefixes, suffixes, involves a series of statistical and linguistic modules, includes **data preparation** (corpora collection and pre-processing), **pronunciation dictionary** (vocabulary selection and dictionary creation), and **language model** estimation.

For the training of the acoustic model, ~50 h of news recordings have been manually transcribed, and for the training of the language model, existing open source text resources are incorporated. The European Parliament speeches^21^, DGT's translation memories^22^, and the *JRC*-ACQUIS c*orpus*^23^ are largest and open multilingual corpora, where we use their monolingual parts for language modeling. Initially, we employed a bilingual crawler developed by Kaumanns (2011) that uses seed webpage URLs, retrieves the HTML-files and processes them offline by a HTML-Parser that separates text from boilerplates; identifies paragraph types; removes inline tags, removes corrupt text portions, etc. Then, the text is divided into sentences by applying syntactic rules. In the sequel, tokenization is applied in order to detach punctuation marks, brackets, and symbols from words. Then, normalization is applied involving harmonization of same token, spelling of numbers, dates, and expansion of abbreviations. Finally, the decomposition step splits compound words into smaller ones and adds them to the lexicon. The preprocessing step eventually transfers all words lower-cased, and each sentence is marked by a start and an end symbol. The module is based on the RWTH-ASR (Rybach et al., 2009) toolkit, which is a speaker-independent, server-based, LVCSR (Ney et al., 1998), and also allows using the open-vocabulary framework, employing continuous density HMMs for acoustic modeling. The supported languages are English and German.

### Semantic analysis

Given that that the data collected and produced by the aforementioned modules are highly distributed, multimodal and heterogeneous, there is the need for their semantic integration that aims at linking, holistic interpreting, assessing all the captured this information. This need is addressed by the semantic analysis module that defines the ontologies for the knowledge representation of annotated content from heterogeneous sources, the infrastructure for the data storage, the reasoner and the decision support system that reasons effectively on large amounts of information. The platform uses two ontologies, namely PROTON and DOLCE, for semantic representation of the relevant information. These ontologies offer the possibility for cleaning statements (the unnecessary ones), their connectivity to linked open data, such as DBPedia, and the availability for applying an effective reasoning mechanism. The infrastructure relied on the OWLIM^24^ repository.

On top of the semantic representation, four types of semantic reasoning techniques are considered, namely the hybrid reasoning, parallel multi-thread reasoning, SPARQL-MM, and GeoSPARQL. **Hybrid reasoning** is critical for scenarios where a target-oriented reasoning mechanism performs on a large amount of data, involves multiple ontologies, and it is popular in semantic data integration and decision support systems. The **parallel multi-threaded reasoning** is able to reduce the loading times from 50 to 200%, when compared to a traditional loading having a serial inference engine. A SPARQL extension, namely **SPARQL-MM** enables reasoning and query answering over media files, and finally, **GeoSPARQL** is able to express geospatial constraints in a powerful language. For the purposes of journalistic usage scenarios, a Decision Support System (DSS) and a Recommendation engine (RE) are developed, aiming to provide semantically enriched information about their desired topics of interest.

More specifically, the RE combines four main components, with each one representing a different aspect of the recommendation objective: (i) relevance of an article with respect to the user and his/her momentary context; (ii) popularity of an article with respect to other articles; (iii) collaborative, i.e., popularity among similar peer users; and (iv) freshness, i.e., the recency of the proposed articles. The above components enable the ordering of a list of news articles, based on weight parameters that determine the effect of each component on the final outcome, and the top n results are returned by the RE as the final outcome. With respect to the DSS of our platform, it belongs to the category of Data-driven systems, which focus on accessing and manipulating large amounts of data. It compares statistical indicators in many factors that need to be considered in a topic of interest and as a result, the users have access to relevant information gathered in one place and supported by charts and diagrams, are able to base their decisions on solid facts rather than speculations.

Semantic reasoning increases the amount of recognized entities by the NER service and, the recommendation engine is expected to obtain more clear and accurate results. DSS and the RE are implemented on top of GraphDB^25^, which is an RDF triplestore. The hybrid search is based on three different types of data retrieval mechanisms—full-text search, faceted search, and concept search. GraphDB connectors have been used for their implementation.

## Delivery layer

The **User Interface (UI)** includes four sections, namely the search section, the results section, the semantic analysis section and the portfolio analysis section, which are described in detail as follows:

**Search section:** With a simple selection of keywords and filtering criteria, the user can make a textual search. The available search methods are the following:

- **Main Semantic Search:** This is the basic search for relevant content using some keywords.
- **Hybrid Search:** When the user starts writing the query, some entities and concepts are suggested in real time with an auto-completion mechanism. Even if some entities and concepts are selected, additional keywords can be used to complement the query.
- **Multimedia Search:** This search method deals with the retrieval of articles, which possess at one or more multimedia elements, such as image, audio, or video.

**Figure 2:**
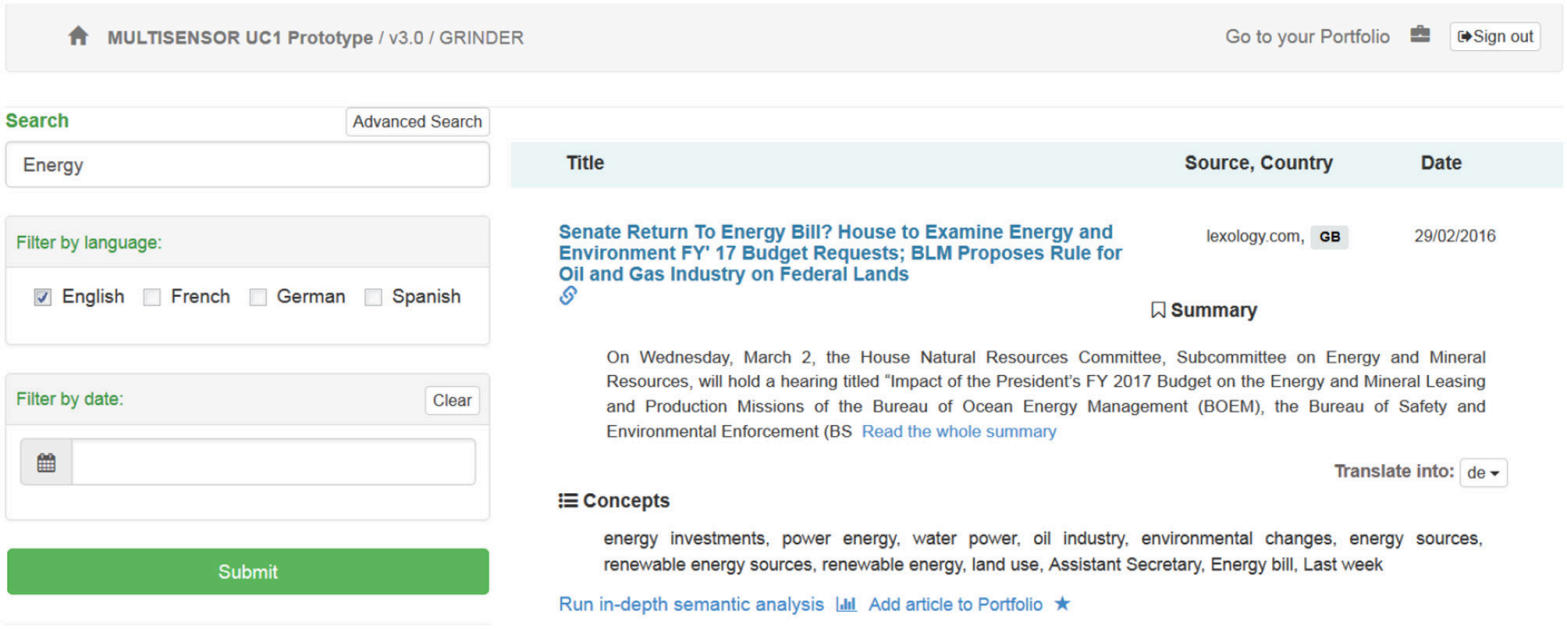
Results page (Header, Advanced search and results listing).

**Results section:** The results view has a dynamic header and a static footer. The header of the page displays user-related information and depends on the status of the user. A non-authorized user will have access to authorization dialog, while an authorized one will be able to access his “Portfolio.” On the left side of the results page, advanced search features are available (a field that supports hybrid search). Search can be done on full text, entities or both. Additionally, filters like search language, and date are also available, as shown in Figure 2.

**Figure 3:**
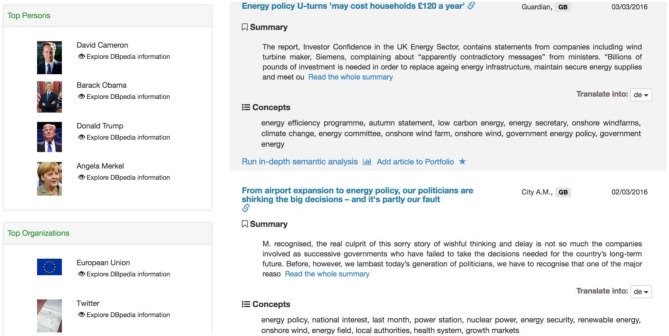
Results page (Entities and results listing).

Search-related entities are also displayed on the left side of the results page. When someone clicks on an entity, it is added to the search query, which can then be used to modify the desired search. Each entity has a link to DBpedia to obtain more information about it. An example is shown in Figure 3.

The right side displays the following information:

- **Context:** Basic elements of an article, such as title, source, etc.
- **Summarization:** Summary as provided by the summarization service.
- **Translation:** Translated summary to one of the available languages.
- **Run in-depth semantic analysis:** Displays the semantic page view.
- **Portfolio creation:** The addition of a news item to the Portfolio section for further analysis.

**Figure 4:**
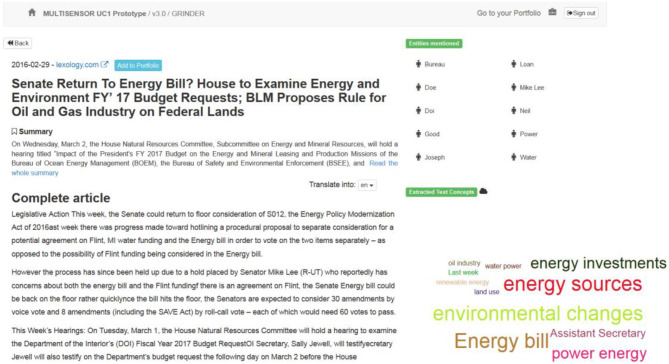
Semantic analytics page (textual dimension).

**Semantic analytics section:** The “Run in-depth semantic analysis” option on the right side of the results page provides a link to a more deep analytics page (Figure 4), which displays more information extracted from the text of an article (a cloud of specific concepts, NEs and related articles). In addition, there is a link to add an article to the Portfolio, for further analysis.

**Figure 5:**
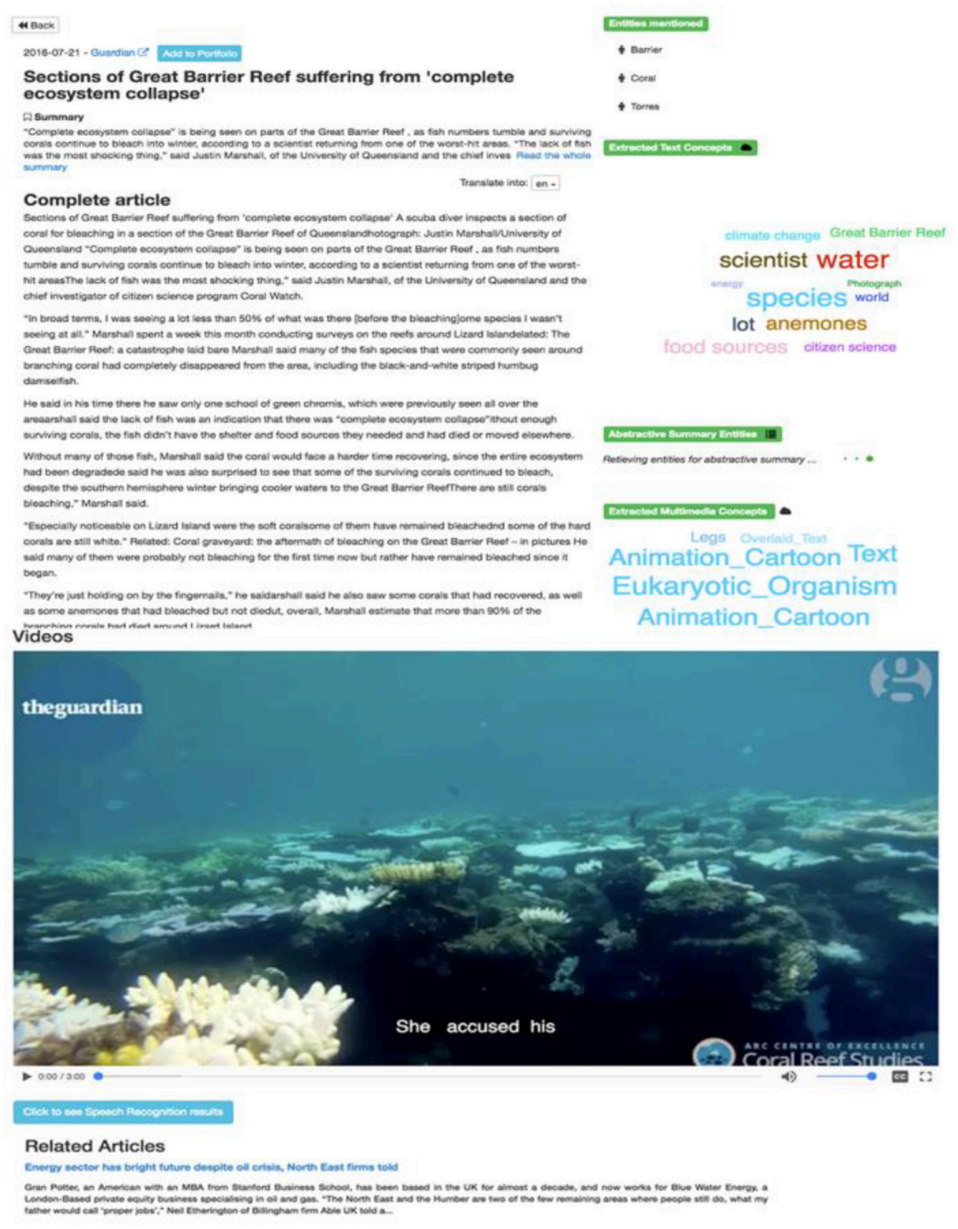
Semantic analytics page (multimedia dimension).

Regarding multimedia content, the semantic analytics page (Figure 5) contains the video player of the multimedia element and the detected multimedia concepts, displayed as a key cloud. The ASR transcript is displayed as subtitles to the video.

**Figure 6:**
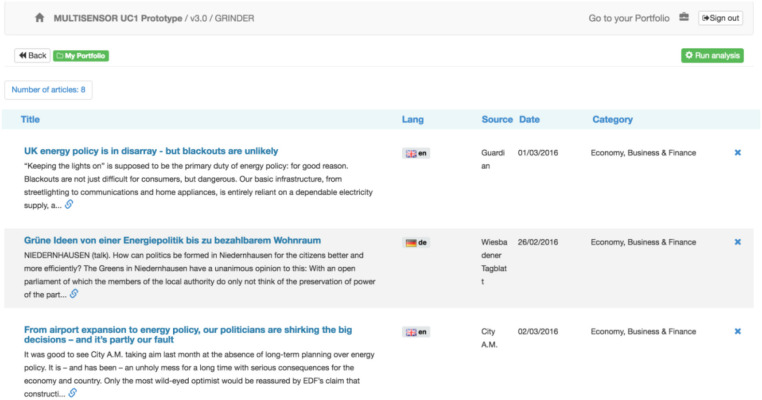
Portfolio home page with selected articles.

**Portfolio analysis section:** During the search process, any article can be added to the “Portfolio” for further analysis (Figure 6). The “Portfolio” is reachable by the “Go to portfolio” button on the top left corner of the header.

**Figure 7:**
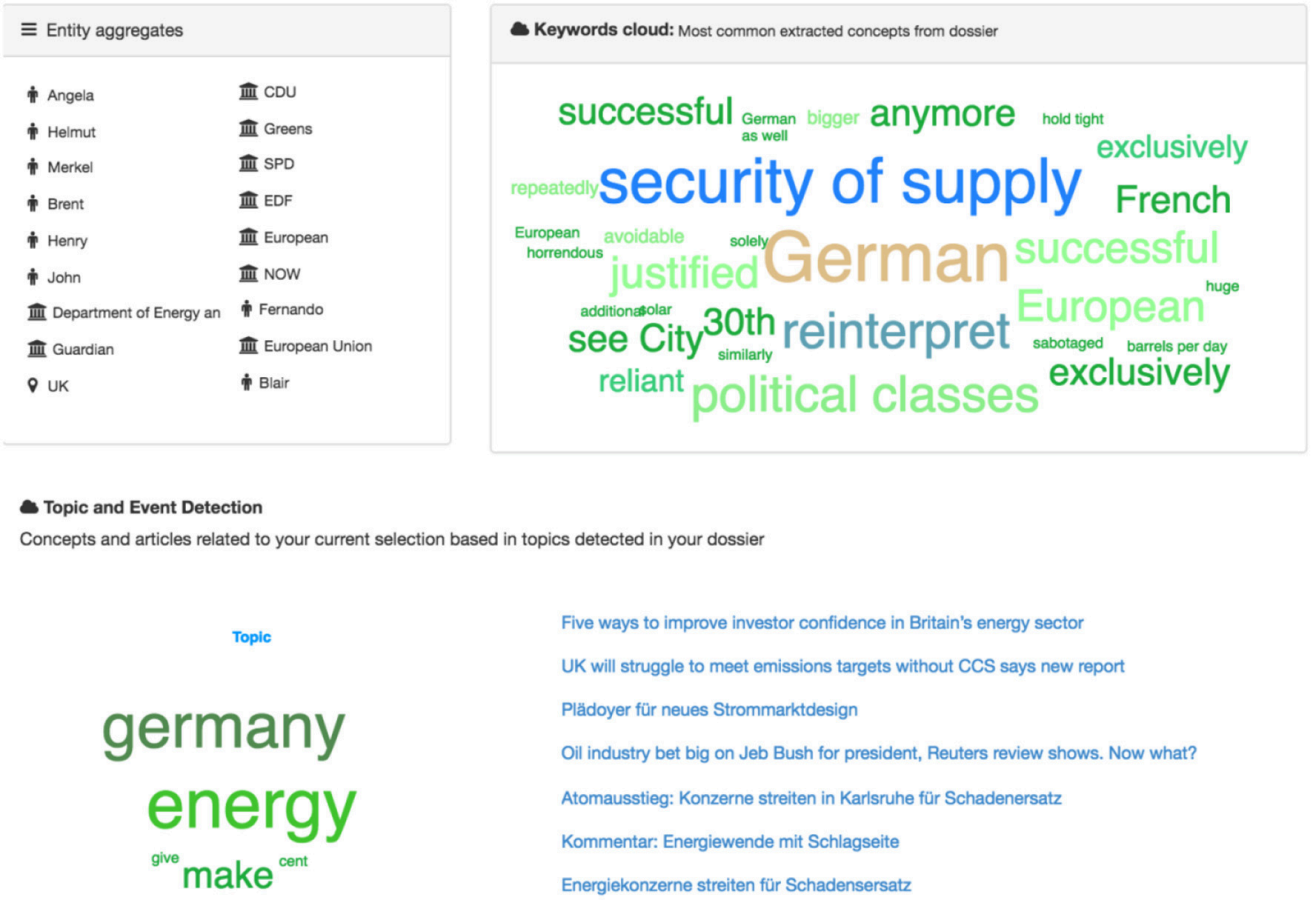
Aggregated Portfolio view.

By clicking the “Run analysis” button, the aggregated analytical view of the Portfolio content can be generated (Figure 7), where the entities, the most frequent words, the extracted topic and similar articles that the topic from this analysis contains, can be seen.

- **Cloud of tags:** A graphical summary of the folder content.
- **Named Entities:** All NEs that appear in the folder.
- **Topic and event detection:** Extracted topics from the Portfolio articles are displayed.

Firstly, the named entities of all documents present in the Portfolio are aggregated. The same approach is followed for the specific concepts. Secondly, the results of the topic and event detection service are aggregated and displayed. For this, the labels of the clusters are shown in the keyword cloud and the list of related articles is provided on the right side of the page.

## Infrastructure

The infrastructure used for hosting the platform consists of two servers, namely mscrawler1 and msgrinder1. Both servers run Ubuntu Linux 14.04.1 LTS (“Trusty”) on x64 architecture. More specifically, mscrawler1 is used for hosting the news articles crawler (described in section News Articles Crawler), dedicated to crawling targeted sites using a combination of Hadoop, Nutch, and HBase. Mscrawler1 has the following specifications:

- 1x x64 core.
- 3.75 GB Ram.
- 32 GB local SSD storage (ext4).
- 100 GB EBS SSD storage (ext4).

The second server, called msgrinder1, is used for hosting the distillation (section Distillation Layer) and delivery (section Delivery Layer) layers of the platform. Msgrinder1 has the following specifications:

- 16x x64 core.
- 122 GB RAM.
- 300 GB local SSD storage (xfs).
- 100 GB EBS SSD storage (ext4).

## Evaluation

The proposed framework is evaluated along two dimensions; system-oriented, which involves the technical evaluation of each module separately by using specific metrics like precision and recall and user-centered focusing on the end-user needs. Since there is no other similar system we cannot directly compare our results with another platform.

### User-centered evaluation

This user-oriented evaluation is centered on the idea of defining a realistic scenario for the user, called a simulated work task situation. According to evaluation best practices, the methodologies used to evaluate the added value and impact of the user tests will be executed following established **usability**, **user engagement**, and **interaction** design benchmarking method. By the term “sability” we refer to the extent to which a product can be used by a particular user to meet particular goals with satisfaction, effectiveness, and efficiency in a given usage scenario. The quality of user-experience that highlights the main assets of the interaction with the application defines user engagement (Attfield et al., 2011). Interaction design refers to the design of a specific user interface and to how this interface facilitates the use of the application itself. Following these definitions, the overlaps between usability, user engagement, and user interaction are obvious. Thus, in order to simplify the evaluation approach and maintain a more simple terminology, we will consider “usability” (Barnum, 2011) as the generic term that stands for the general concept of user-centered evaluation including the testing of user engagement and interaction. ISO 9241-11 formally defines **effectiveness** (to which level the user is able to achieve his/her goals), **efficiency** (to which level of effort the user has to invest over the achieved accuracy), and **satisfaction** by working with the system.

In our evaluation methodology we also distinguish between formative and summative testing. **Formative testing** is carried out along with development stage and targets at identifying problems that require to be fixed. It is less about metrics or statistics, but about finding out what works best for users. **Summative testing** verifies whether the final outcome satisfies the user needs. Despite the differences between formative and summative testing in general, the evaluation process is similar. Test persons were given specific tasks that they had to perform with the system in order to assess its amenities and shortcomings.

Given, the proposed framework is in final status, the type of testing that is realized is summative. The main questions that need to be answered are the following:

- To which extent does the system support the user in fulfilling a specific task that is typical for his day-to-day work (task-related evaluation)?
- To which extent does the system meet the requirements that have been formulated with regard to system usability?

The participants involved in the evaluation were 35 professionals, with 40% journalists, 37% researchers, and 23% participants of other professions. For this purpose, dedicated evaluation sessions were organized, during which the participants had access to the platform and were able to perform the specific tasks of the evaluation methodology. Ninety-five percentage of the test participants were able to complete the complex task that was given to them. A scenario was created (for the evaluators) that is able to make them judge all functionalities that are included in the platform. The duration of the task was ~30 min and for personal reasons 5% of the evaluators did not complete the evaluation procedure.

The questionnaire was validated and approved by DW, a large public broadcaster, which served as a partner of the MULTISENSOR project, as well as co-authors of this paper. Special attention was given to any ethical issues regarding the formulation of the questionnaire, approved within the DW organization. The questionnaires may be found online^26^.

### Task-related evaluation

The test participants were given the specific task to create a portfolio (dossier) consisting of at least five articles relevant to the topic “Energy policy in the UK and the US.” They were asked to explore all available functionalities that were provided by the system. After having completed the portfolio, test persons were asked to run a “portfolio analysis” and to assess its quality. The main question throughout the evaluation was whether a specific feature (i.e., module or functionality) was useful for quickly deciding on the relevance of an article.

#### Content summarization

**Figure 8:**
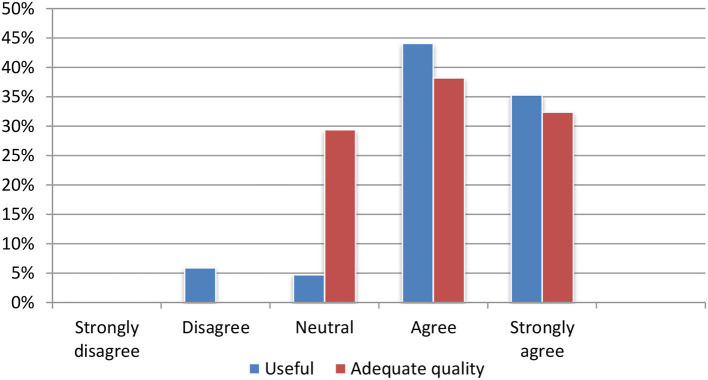
Evaluation of the summarization module.

The content summarization module produces summaries, which are, according to user feedback, perceived as particularly useful. Nearly 90% of all test persons agreed or strongly agreed that the summarization tool was useful for quickly deciding on the relevance of an article. Seventy percentage assessed the quality of the summaries as adequate (Figure 8).

#### Machine translation

**Figure 9:**
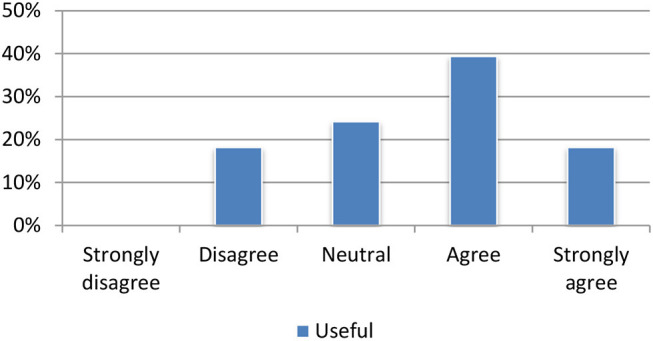
Evaluation of the machine translation module.

The translation module received mostly positive feedback. Most of the test participants agreed or strongly agreed that the translations were useful for assessing the relevance of an individual article (Figure 9).

#### Named entities and textual concepts extraction

**Figure 10:**
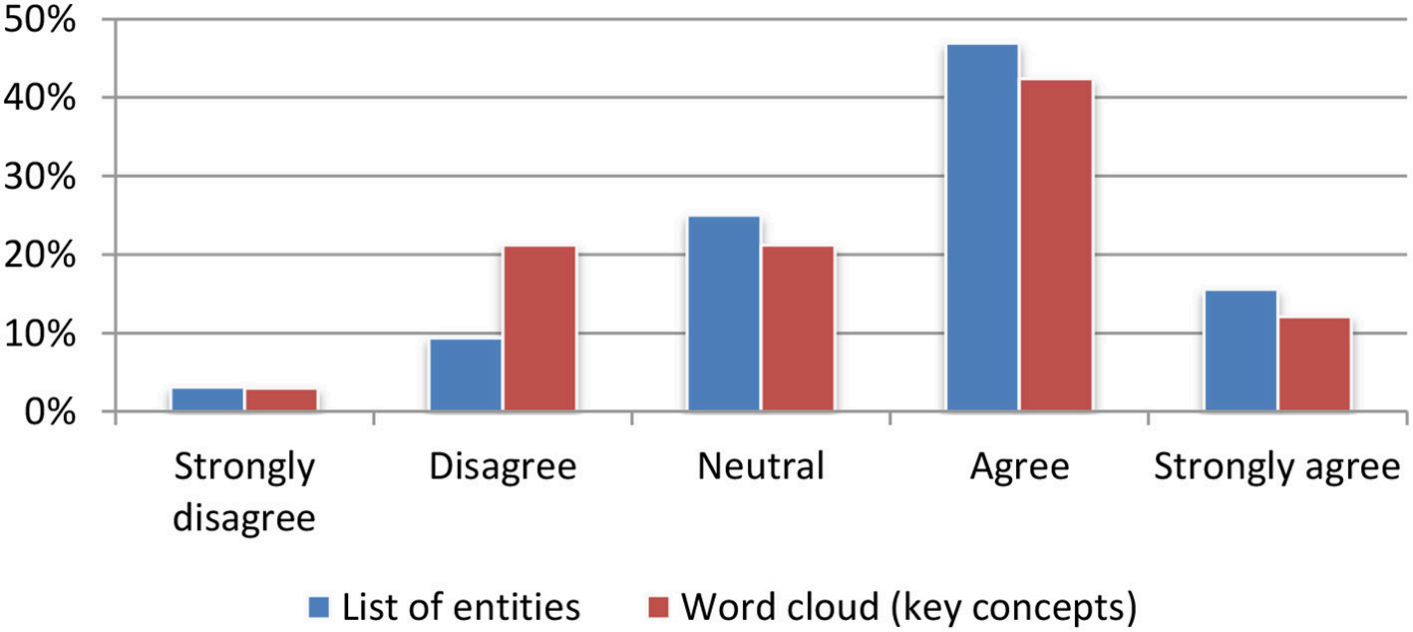
Named entities and textual concepts extraction for single item.

**Figure 11:**
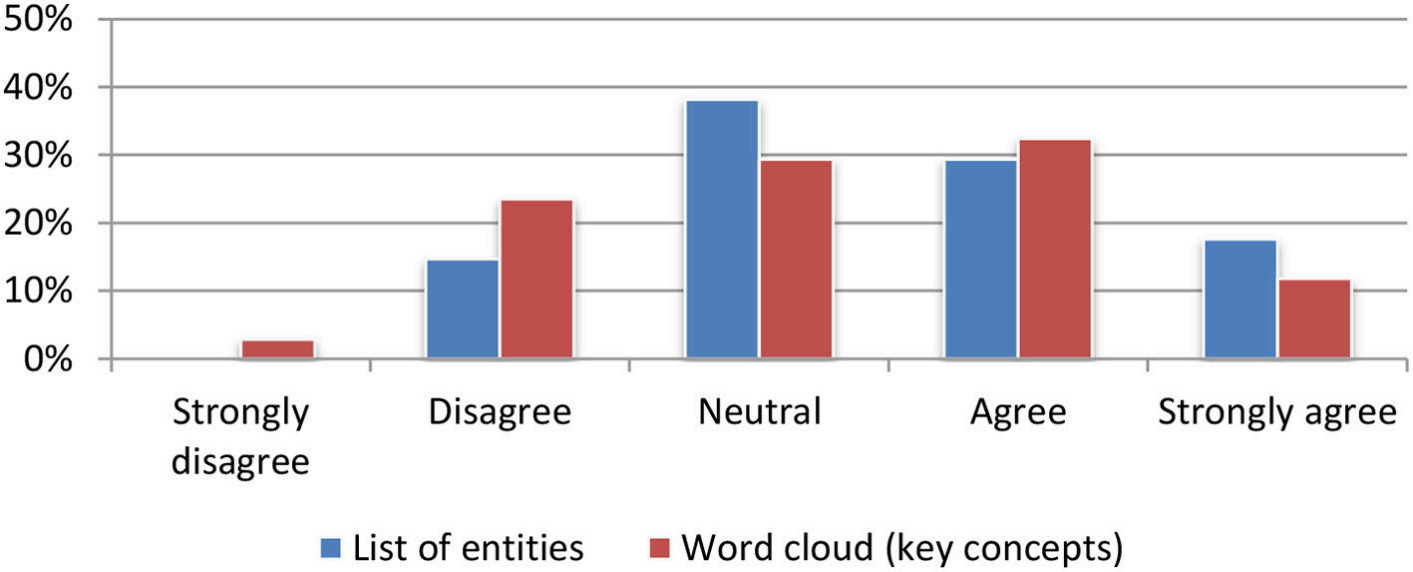
Named entities and textual concepts extraction for aggregated items (portfolio).

Named entities and textual concepts were assessed based on their usefulness for quickly deciding on the relevance to either a single article (Figure 10) or to a set of articles stored in the portfolio (Figure 11). The results were mainly positive with a small preference for the list of named entities.

#### Topic and event detection

**Figure 12:**
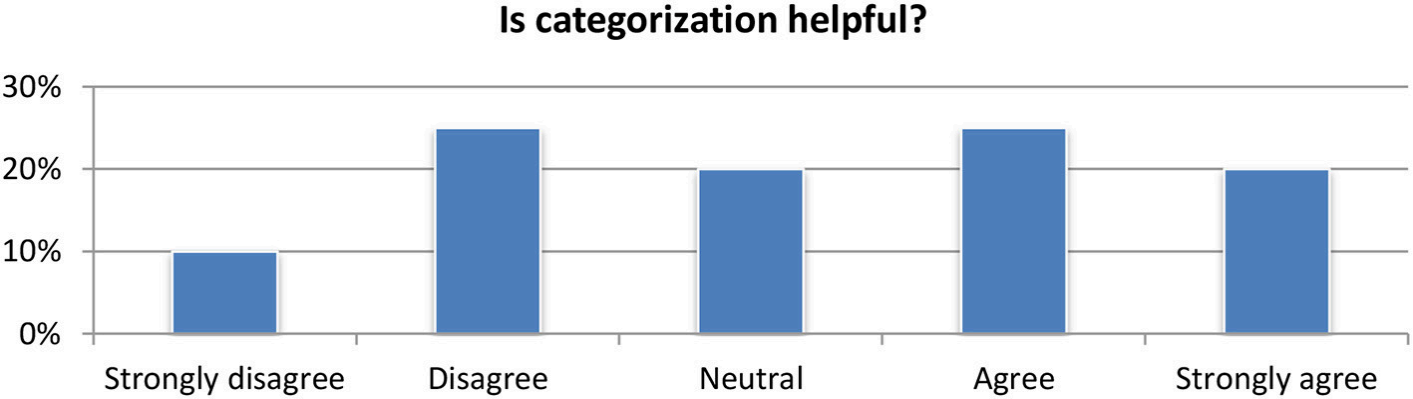
Topic-category classification module evaluation.

**Figure 13:**
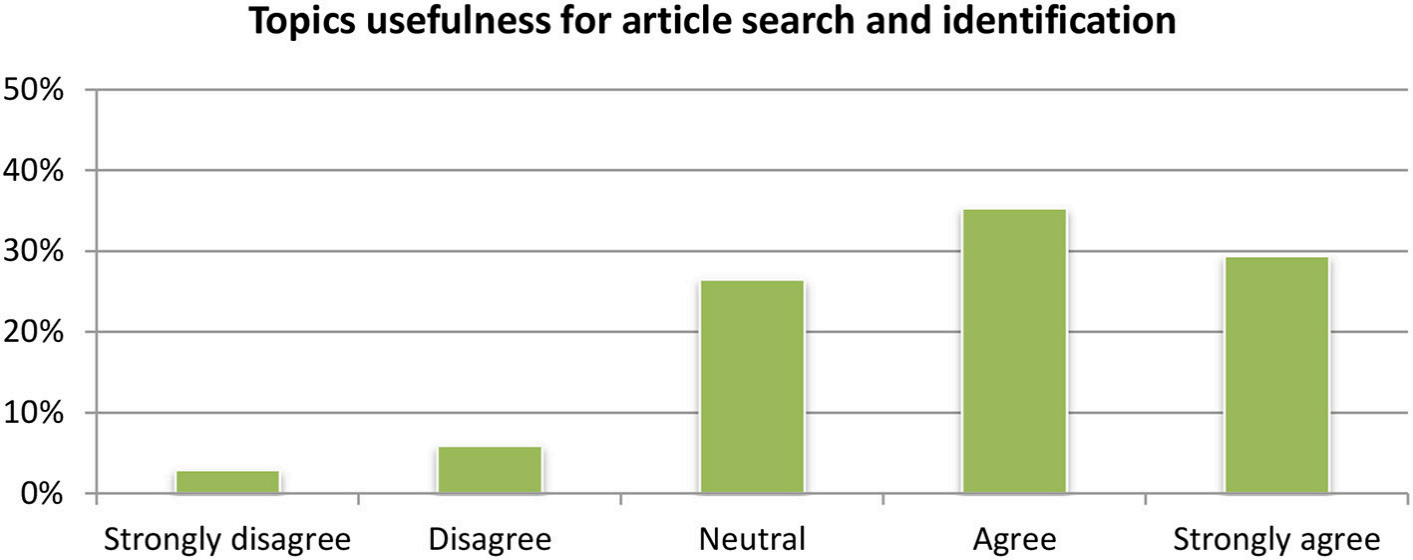
Topic-event detection module evaluation.

Regarding the category classification (Figure 12), the vast majority of the users (65%) were satisfied by the module and considered it as useful (Figure 13). Regarding the detection of topics, the module is available in the portfolio section where it clusters all the documents stored in the portfolio by their concepts and named entities. Compared to the aggregated word of key concepts and named entities, the clustering of articles based on related topics was considered very is relevant for expanding the research and identifying new articles by the vast majority of users.

#### Multimedia concept detection

**Figure 14:**
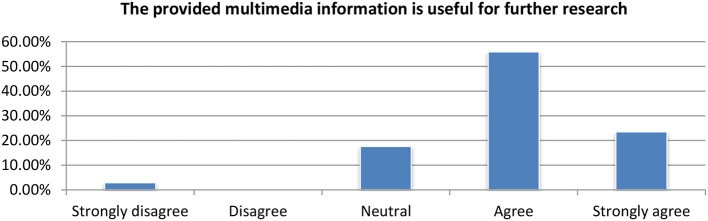
Evaluation of the multimedia modules.

Concept detection using multimedia as well as the ASR module where evaluated under a single question asking whether the multimedia information was useful for further research or not. The responses where very positive since the vast majority of users found it very useful (Figure 14).

### Usability testing

The platform was evaluated as a whole by assessing its effectiveness, efficiency and satisfaction with regard to the integrated modules and its general performance in supporting a user with a typical task.

#### Effectiveness evaluation

**Figure 15:**
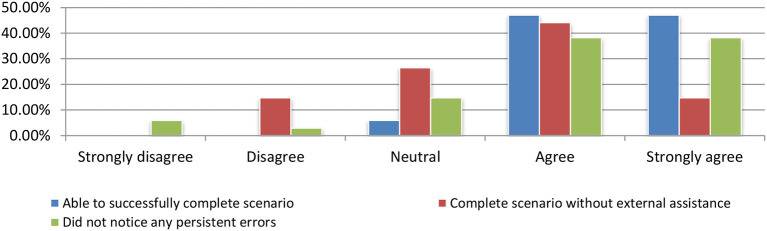
Effectiveness evaluation.

Nearly all test participants were able to successfully complete the tasks that they had been given and perceived the system as effective (as shown in Figure 15) by answering the following questions: (a) I was able to successfully complete the scenario, (b) I could finalize the scenario without additional support, and (c) I did not realized any errors while using the application.

#### Efficiency evaluation

**Figure 16:**
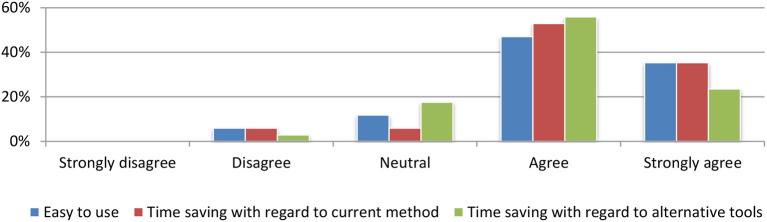
Efficiency evaluation.

After the assessment of the effectiveness of our system, users were requested to evaluate its efficiency. The question was how user-friendly the platform was and the overall time spent for them to perform the tasks. The results revealed a very promising outcome, as shown in Figure 16.

#### Satisfaction evaluation

**Figure 17:**
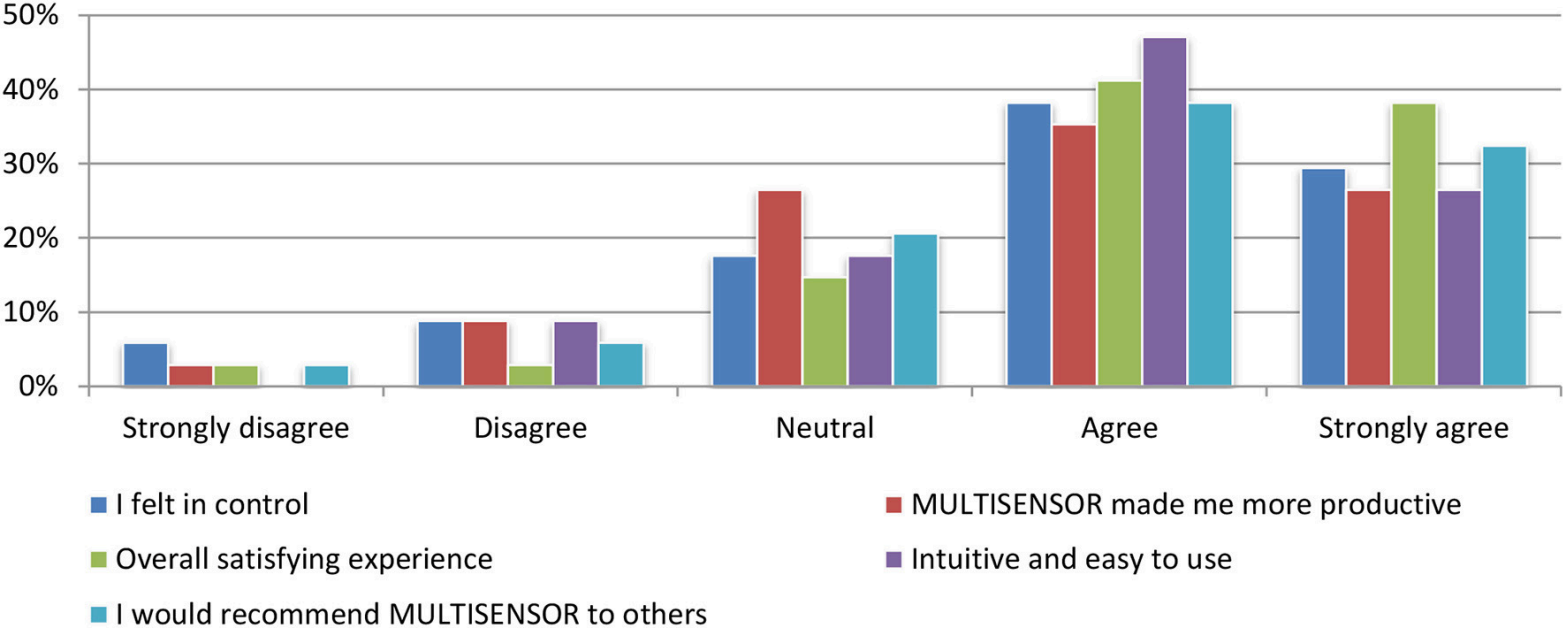
Satisfaction Evaluation.

More than 75% of all test participants perceived the interface as intuitive and assessed the use of platform as an overall satisfying experience. In addition, a clear majority said that they felt in control (67%) and more productive (62%) when using it. A further and even 70% would recommend the system to others. Figure 17 depicts an overview of all queries related to satisfaction with the system and the user responses.

Test persons made a number of very diverse comments ranging from detailed feedback on individual modules to suggestions for how to improve the user interface. Furthermore test persons were asked which functionality of they perceived as “most promising and suitable” for further development and subsequent exploitation and although most of the tested modules were mentioned at least once, “summarization” was clearly the functionality that got most of the votes. Overall, evaluation in the journalistic use case has shown very positive results. With regard to nearly all individual functionalities that have been tested, a clear majority of up to 75% of test persons agreed or strongly agreed with the usefulness for fulfilling a very specific task of the respective functionality.

### System-oriented evaluation

#### Content summarization

The system-oriented evaluation of the content summarization module has been performed by the comparison of the summaries of 261 texts, as they are generated by SUMMA, to their corresponding human-authored summaries. The annotations in the original text documents of a second corpus have then been imported as GATE annotations and, together with the corresponding IDF table obtained from the first corpus, we run a SUMMA-based pipeline for single-document summarization. Both corpora have been processed using a specific pipeline, set up for this evaluation and has four modules: linguistic pre-processing with Mate tools^27^, deep dependency parsing with Mate tools (Björkelund et al., 2010), entity linking with Babelfy REST service, and FrameNet-based n-ary relation extraction with our own module. The evaluation metric is unigram ROUGE (Chin-Yew, 2004) which compares lexical overlap of unigram vectors from sentences in the summary to be evaluated to the vectors of sentences in the gold standard summary. For our evaluation we run the SUMMA pipeline three times using features and IDF tables for the metrics 1, 2, 3, and 6 of the unigram ROUGE. The evaluation of the content summarization module resulted to 0.598 for the metric 1, 0.581 for the metric 2 and 0.579 for the metric 6.

#### Machine translation

The quality of the MT module is mainly assessed by automatic metrics, which include several tools that assess automatically the quality of the translation quality such as BLEU (Papineni et al., 2002), NIST^28^ and METEOR (Denkowski and Lavie, 2011). These tools measure the similarity of the MT output with one or several benchmark translations. Moreover, there is the manual evaluation which involves manual comparison two MT outputs and humans decide which one is better. Within our platform and due to the considerable human effort required by the manual evaluations, we have opted for automatic metrics and specifically for BLEU. The test set consists of 3,000 sentences for each language direction. Table 2 presents the results of the MT development cycles using the BLEUscores for several improvements realized on the module including: (S1) better homogenization of the training corpus which involves consistent true-casing and tokenization, and reduction of spelling variants to one “standard term,” (S2) reduction of the amount of unknown words by either enriching the phrase tables or by adding translation for unknown words or by retraining the model, and (S3) tuning the model parameters for better quality, which involves setting up a development set that is domain and application-specific as possible.

**Table 2:** MT evaluation results measured in BLEU.

	**S0**		**S1**		**S2**		**S3**	
	**TC**	**LC**	**TC**	**LC**	**TC**	**LC**	**TC**	**LC**
bg-en	22.48	24.04	24.87	25.99	25.99	26.01	27.11	28.99
de-en	18.09	18.81	18.81	18.90	18.90	18.91	18.80	19.57
es-en	19.73	20.33	20.66	20.93	21.00	21.02	23.92	24.95
fr-en	19.64	20.29	20.28	21.34	21.54	21.99	23.31	24.13
en-de	14.50	14.71	14.71	14.81	14.81	14.81	14.90	15.13
en-es	23.19	23.97	23.90	23.91	23.93	23.94	23.19	23.97
en-fr	23.54	24.08	24.05	24.01	24.05	24.08	23.54	24.08

#### Named entities

To provide a system-oriented evaluation for the NE recognition module, we extracted from our repository the following test sets: 200 documents per language, and per usage scenario; of these, 20 documents were randomly selected as test documents. To achieve a more realistic evaluation scenario, the documents were not further cleaned or processed, so they contain spelling, formatting, and other errors. This resulted in 60 documents per language, 20 per use case scenario. We present the test systems which were used for our experiments and the corresponding evaluation results.

##### Test systems

To directly compare the performance of existing versions (V1506) and the current version (V1510), we test them separately in two directions; first, a lexicon-based (V1506L/V1510L); second, a grammar-based, taking also contexts into account (V1506G/V1510G), obtaining four systems for testing:

- V1506L basic lexicon, only lexicalized NEs recognized
- V1506G also contextual indicators for NEs are used
- V1510L domain-specific lexicon, only lexicalized NEs recognized
- V1510G domain-specific lexicon, using also contextual indicators for NEs

##### Results

The results are presented in Table 3 and our observations are summarized as follows: The version V1510 has significantly better results in both lexicalized (V1510L) and grammar (V1510G) versions than V1506. Domain adaptation on the lexical level, by lexicalizing domain-specific named entities, is a significant contribution to the recognition quality in all test documents, observing an average absolute increase of 0.4 in recall and precision performance measures. Moreover, we observe that the grammar in V1506G improves recall but reduces precision when compared with V1506L, where we conclude that the contextual grammar heuristics introduce a critical amount of noise. This does not occur in the case of V1510, where the grammar-based contextual analysis improves both recall and precision, due to the refined contextual analysis of our system. We also present the evaluation on a specific entity: “person names.” The results are shown in Table 4, where it is observed that our contextual analysis increases recall and precision more than any other NE type. An error analysis has shown that a main source noise generation is the appearance of homographs between common nouns which are capitalized in headings and proper names.

**Table 3:** Evaluation of the system versions by domain.

**Domain**	**Energy**	
**System**	**Recall**	**Precision**
V1506L	0.31	0.58
V1506G	0.37	0.45
V1510L	0.71	0.76
V1510G	0.74	0.79

**Table 4:** Evaluation of NE-type “Person names”

	**Domain-specific**		**Total**	
	**Recall**	**Precision**	**Recall**	**Precision**
V1506L	0.15	0.08	0.29	0.13
V1506G	0.57	0.30	0.61	0.33
V1510L	0.47	0.34	0.54	0.34
V1510G	0.79	0.50	0.78	0.45

#### Concept extraction

To evaluate the concept extraction task, three annotators annotated a text composed of 20 sentences for the journalistic scenario that involves energy policies (Table 5). To evaluate the system and observe the impact of merging the two approaches, we measured separately the performance of the statistical and BabelFy approaches. Then, we measured the performance of the final system. Table 6 shows the precision and recall of the two different approaches and of their merge (“Hybrid System”).

**Table 5:** Number of terms annotated for each use case and number of indexed documents.

**Domain**	**Number of documents**	**Number of indexed splits**	**Annotated terms**
Reference corpus	21,994	43,308	-
Energy policies	1,000	1,565	80

**Table 6:** Precision, recall and f-score of different approaches.

	**Precision (%)**	**Recall (%)**	**F-score (%)**
Statistical approach	28.0	97.3	43.5
BabelFy approach	36.2	74.68	48.8
Hybrid system	48.3	70.9	57.4

It can be observed that the hybrid system increases the precision between 14 and 25%, while the recall decreases between 7 and 24%. To measure whether the increase on precision compensates for the loss of coverage, we computed the F-score. F-score shows that the hybrid system is 7% over the score of the BabelFy approach and 13% above the statistical approach. These results reflect the processing of all terms provided by both tools and only after filtering out the extreme cases.

#### Category-based classification

The category classification module was trained and validated on a dataset of 12,073 news articles that were manually annotated and classified to 6 topics mentioned in section Topic and Event Detection. Specifically, a random balanced split was applied within each class and 2/3 of the cases were used for training, while the rest for validation and for estimating the performance of the classification models. Regarding the parameters set, to obtain the best parameter values of the word2vec model, we applied a grid search approach, coupled with cross-validation for the dimensionality of the vectors and the width of the context window. The obtained optimal values were 200 for the number of dimensions and 12 for the context window width. Regarding the number of RF trees, our tuning has shown that after 1,000 trees, the OOB error reached an equilibrium, thus the number of trees is set to *T* = 1,000. Table 7 contains the results from the application of RF to each text representation and the results from our late fusion approach that combines the RFs using the estimated OOB error. We observe that the late fusion scheme outperforms the baseline approaches.

**Table 7:** Classification results.

** 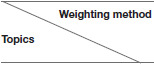 **	**N-gram**	**Word2vec**	**N-gram + Word2vec**	**Weights assigned to each representation**	
	**F-score (%)**	**F-score (%)**	**F-score (%)**	**N-gram**	**Word2vec**
Economy/Business	85.6	84.6	86.5	0.52	0.48
Health	61.8	54.2	60.1	0.444	0.556
Lifestyle/Leisure	86.7	82.0	86.8	0.505	0.495
Nature/Environment	86.2	83.2	86.1	0.507	0.493
Politics	62.1	69.9	71.6	0.431	0.569
Science/Technology	78.4	71.1	76.9	0.539	0.461
Macro-average	76.8	74.2	78.0		
Accuracy	83.3	79.8	83.6		

#### Topic-event detection

The system oriented evaluation of the topic-event detection module is done by first extracting 10,476 webpages in total, targeting at multilingual topic detection using the extracted NEs and concepts. The module runs firstly the DBSCAN-Martingale to estimate the number of topics and then assigns the news articles to topics using LDA. The topics are “energy crisis,” “solar energy,” and “energy policy,” as they are retrieved from our central news repository. For each news article we know the topic in which it belongs to and this annotation is used as ground truth. In order to evaluate the clustering of the retrieved news articles, we compare the average precision (AP), within each cluster (topic) against the AP of other popular methods to estimate the number of clusters (Gialampoukidis et al., 2016). The results of the AP scores per query and the mean AP scores (MAP) per method are shown in Table 8. We observe an improvement of **9.65%** in MAP, when our method is compared to the second largest MAP value.

**Table 8:** Average Precision (± standard deviation) and comparison with several baselines.

**Index + LDA**	**Energy crisis**	**Energy policy**	**Solar energy**	**MAP**
Duda	0.4498 ± 0.0671	**0.5534** ±**0.0457**	0.4484 ± 0.0067	0.4839
Pseudo t∧2	0.4498 ± 0.0671	**0.5534** ±**0.0457**	0.4484 ± 0.0067	0.4839
Hartigan	0.5938 ± 0.0502	0.5336 ± 0.0375	0.5961 ± 0.0347	0.5745
Silhouette	0.5786 ± 0.0425	0.5371 ± 0.0357	0.5961 ± 0.0347	0.5706
Dunn	0.5786 ± 0.0425	0.5371 ± 0.0357	0.5961 ± 0.0347	0.5706
SDindex	0.3541 ± 0.0181	0.3911 ± 0.0033	0.4484 ± 0.0067	0.3979
NbClust	0.5786 ± 0.0425	0.5371 ± 0.0357	0.5961 ± 0.0347	0.5706
HDBSCAN-EOM	0.4498 ± 0.0671	0.3911 ± 0.0033	0.5375 ± 0.0446	0.4595
DBSCAN-Martingale	**0.7691** ±**0.0328**	**0.5534** ±**0.0457**	**0.6073** ±**0.0303**	**0.6433**

*Bold values show the maximum performance per method*.

#### ASR evaluation

Given a ground truth transcription (what was actually said) and the ASR output, the similarity in words between these two sets is usually measured by the word error rate (WER), calculated by the open source tool NIST sclite^29^. The Word Error Rate (WER) is the most commonly used performance measure of automatic speech recognition systems and not by the accuracy. The reason is that the accuracy cannot take into account word insertions, deletions, and substitutions of words and, therefore, it is weaker than WER evaluation measure in ASR tasks. For the German language, the overall WER without post-processing was 19.3% while with post-processing the overall WER is reduced to 15.8%, which is an absolute improvement of 3.5%. The dataset involved contains 100 news videos and audios collected from Deutsche Welle for both English and German, comprising 4.9 and 4.7 h of recording, respectively. The recordings were then manually transcribed and the results crosschecked by 2 other annotators for correctness. Twenty-five of the recordings were from the domain energy policies, the rest from general news. The recordings represent a broad mix of speakers (moderators, interviewees) and audio conditions (background noise, indoor, outdoor, music, etc.). This corpus was taken as a benchmark for the evaluation of the recognition accuracy of the ASR component and is presented in Table 9.

**Table 9:** Evaluation corpus used as gold standard.

**Language**	**No. of Recordings**	**No. of Words**	**Audio length**
English	100	40,380	4.9 h
German	100	39,100	4.7 h

The resulting WERs vs. the baseline versions for English and German are shown in Table 10.

**Table 10:** Improvements of advanced ASR vs. baseline systems.

**Language**	**ASR System**	**WER**	**Improvement**	
			**Absolute**	**Relative**
English	Baseline	16.7%		
English	Advanced	15.1%	1.6%	9.6%
German	Baseline	15.8%		
German	Advanced	14.4%	1.4%	8.9%

#### Multimedia concept extraction

The multimedia concept extraction module is evaluated both in the concept and the logo extraction sub-problems. As a common practice, in case a very domain-specific is not available publicly online, the overall procedure for creating the training datasets involves the collection of relevant-to-a-concept images downloading public and open images from Bing, Flickr, and Google, using their corresponding official APIs, and a manual annotation stage follows for verification of the retrieved images. Some irrelevant images are also added to create a set of irrelevant images that do not include any reference to the considered logo or concept. Different testing datasets were developed for the different descriptors given that the concepts targeted differ significantly. The testing dataset was created by collecting 20 relevant images for each logo concept and 200 irrelevant images, thus resulting to 300 images. Regarding the considered logos, the results are shown in Table 11.

**Table 11:** Evaluation results using F-score measure.

**Concept name**	**F-score**
EnBW logo	0.625
E-On logo	0.687
Nuclear energy logo	0.857
RWE logo	0.687
Vattenfall logo	0.709
Macro-average	0.713

The testing set for the DCNN-based models that involves more general concepts such as Wind turbine and solar panel are 106 video extracts from news reports, provided by the DW data repository and collected from Youtube including reports from well-known news agencies (e.g., BBC). The dataset and the ground truth video annotations are available online^30^. In Table 12, we report the classification performance results for the general concepts and provide at the same time a comparison with the SIFT-based descriptors. We notice that the models trained on DCNN features achieve a good performance considering how demanding the dataset is due to the few available positive examples for each concept.

**Table 12:** Evaluation results for the two concept/event detection frameworks.

**Concept name**	**Local Features (SIFT/ RootSIFT)**	**DCNN features**
Outdoor factory smoke	0.111	0.554
Wind turbine	0.460	0.826
Solar panel	0.483	0.863
Lattice tower	0.545	0.693
Construction workers	0.212	0.589
People protesting	0.382	0.711
Speaking to camera	0.647	0.944
Fire	0.606	0.647
Airplane flying	0.267	0.700
Macro-average	0.415	0.725

## Conclusion

In this paper, we presented a platform integrating multimodal analytics techniques, focusing on journalists as end users and, in general, targeting the media community as interested parties and stakeholders. Our platform supports the management of large streams of real-time and heterogeneous information, offering multiple functionalities which join forces for the journalistic work. The presented platform automatically crawls and indexes multilingual and multimedia information, text is automatically summarized and can be translated into the language of the user. Named Entities and concepts are extracted from both textual and visual content for fast inspection using concept clouds. Moreover, audio is transformed to text for semantic integration with the other sources of information, indexed using a common representation, so as to be delivered to the journalist through a web-based search engine. The platform has been tested in five major European languages. We restrict our analysis and evaluation in these five languages for demonstration purposes and its extension to other languages is one of our future goals.

The evaluation of the proposed platform was performed by several groups of journalists and has shown satisfactory results. The use of such a platform by the journalists could allow them to access large streams of online news content. The delivery of a summary of current trends in the news, from an unknown language to the language of a journalist, enriched with semantic concepts from textual, visual and audio data, supports significantly the journalistic work, allowing journalists to make quicker decisions on articles and topics, covering more news items in less time. In addition, the social impact of the platform should be highlighted, in the sense that cross-validated news articles are produced and news stories from different cultural and linguistic perspectives are presented. Finally, the extraction of high-level information is expected to benefit future additional modules, such as the detection of fake news.

## Author contributions

SV coordinated the preparation of the paper and contributed to the Distillation layer along with AM, IG, DL, IK, GC, and LW led the preparation of the summarization and textual concept extraction. IA contributed to the Harvesting layer. NH and TW led the evaluation part. AB and EJ where responsible for the platform development and system integration parts. BS and VA led the semantic analysis and RB the machine translation module.

### Conflict of interest statement

The authors declare that the research was conducted in the absence of any commercial or financial relationships that could be construed as a potential conflict of interest.

## Footnotes

^1^https://mklab.iti.gr/multisensor/images/f/f9/D8.2_RequirementsUseCasesValidationplan_2014-07-29_v1.1.pdf

^2^http://www.virato.de

^3^http://emm.newsexplorer.eu/NewsExplorer/home/en/latest.html

^4^https://rivva.de

^5^https://mention.com/en

^6^https://www.brandwatch.com

^7^http://www.opencalais.com

^8^https://www.programmableweb.com/company/alchemyapi

^9^http://nutch.apache.org/

^10^http://hadoop.apache.org/docs/r1.2.1/mapred_tutorial.html

^11^http://hadoop.apache.org/

^12^http://hbase.apache.org/

^13^http://www.socialsensor.eu/

^14^http://www.taln.upf.edu/pages/summa.upf/

^15^https://gate.ac.uk/

^16^http://www.iptc.org/site/Home/

^17^http://ffmpeg.org/

^18^http://caffe.berkeleyvision.org/

^19^https://github.com/BVLC/caffe/tree/master/models/bvlc_googlenet

^20^https://www.csie.ntu.edu.tw/~cjlin/libsvm/

^21^http://www.statmt.org/europarl/

^22^https://ec.europa.eu/jrc/en/language-technologies/dgt-translation-memory

^23^https://ec.europa.eu/jrc/en/language-technologies/jrc-acquis

^24^A semantic repository developed by Ontotext, http://www.ontotext.com/owlim/

^25^https://ontotext.com/products/graphdb/

^26^https://www.multisensorproject.eu/wp-content/uploads/2016/11/D8.5_Final_System_EvaluationReport_2016-11-10_v1.0.pdf

^27^https://code.google.com/p/mate-tools/

^28^ftp://jaguar.ncsl.nist.gov/mt/resources/mteval-v13a-20091001.tar.gz

^29^https://www.nist.gov/itl/iad/mig/tools

^30^http://mklab.iti.gr/files/Event_Detection_Dataset_MS.rar
